# A Biopsychosocial Evaluation of Post-Acute Outcome of Patients with Severe Brain Lesions Recovering from Coma: An Exploratory Study

**DOI:** 10.3390/jcm12103572

**Published:** 2023-05-20

**Authors:** Noah F. La Framboise, Etienne Rochat, Karin Diserens

**Affiliations:** 1 Faculty of Biology and Medicine (FBM), Lausanne University, 1005 Lausanne, Switzerland; 2 Institute of Humanities in Medicine, Faculty of Biology and Medicine (FBM), Lausanne University, 1007 Lausanne, Switzerland; 3 Department of Clinical Neurosciences, Lausanne University Hospital (CHUV), 1011 Lausanne, Switzerland

**Keywords:** coma recovery, post-acute phase, biopsychosocial-spiritual, ICF

## Abstract

Currently, very little is known about the holistic outcome of patients recovering from coma. The aim of this retrospective exploratory study was to evaluate the outcomes of patients recovering from coma after care in an acute neurorehabilitation unit with particular focus on their biopsychosocial and spiritual needs in the post-acute phase of recovery. We included 12 patients and evaluated clinical outcome evolution by comparing standard neurobehavioral scores from patient files measured in the acute and post-acute phases. We assessed patient needs using the Quality of Life after Brain Injury scale (QOLIBRI) and classified self-reported complaints mentioned in patient files according to the International Classification of Functioning, Disability and Health framework (ICF). Mean patient evolution was a Level of Cognitive Functioning Scale (LCF)-r increase of 3.33 levels (range = 2); a Disability Rating Scale score (DRS) of −3.27 points (SD = 3.78); a Functional Ambulation Classification (FAC) scale score of 1.83 (range = 5); and a Glasgow Outcome Scale (GOS) median = 0 (Interquartile range = 1). Main patient complaints concerned mental functioning (*n* = 7), sensory functioning and pain (*n* = 6), neuromusculoskeletal and movement problems (*n* = 5), and major life areas (*n* = 5). To conclude, a significant handicap that affects their daily life was present in the post-acute phase in most patients. Complaints involved biopsychosocial and spiritual elements. The neurobehavioral scale results do not necessarily correlate with the subjective representations patients had of their condition.

## 1. Introduction

In the acute phase, a better understanding of the state of a patient’s consciousness can have an impact on their prognosis, influencing major medical decisions such as those involving the potential interruption of life sustaining therapies [1,2]. The acute neuro rehabilitation unit (NRA) of Lausanne University Hospital (CHUV) primarily focuses on the management of patients who are recovering consciousness in the acute phase following coma. It consists of a multi-disciplinary team that is active in developing new clinical tools to improve consciousness diagnosis in individuals with severe brain injury [1,3,4]. After acute phase care and management by the NRA multi-disciplinary team, patients are transferred to different rehabilitation centres for long-term recovery care. At present, very little is known regarding the long-term evolution of patients having benefited from acute management in this unit.

In addition, research regarding post-acute and chronic phases of coma recovery has essentially used an “objective” perspective for assessing functional outcomes. Very few studies have considered a “subjective” approach by evaluating the patient’s view of their condition [5,6], and to the best of our knowledge, no study has investigated coma recovery outcomes using a biopsychosocial and spiritual model.

To respond to a demand from patients and their families, and to improve continuity in the medical journey, the NRA team introduced a post-acute phase follow-up appointment in early 2021. In addition to assessing and serving somatic aspects of their condition, it is known that patients recovering from major neurological damage require attention to the wider aspects of their lives, including the environment in which they live during follow-up [5,7]. Consequently, in these follow-up appointments, two main steps were taken. First, when possible, a social and spiritual interview was carried out. Secondly, to increase the understanding of the patient’s perception of their own situation, a health-related quality of life assessment was undertaken using the Quality of Life after Brain Injury (QOLIBRI) instrument [8]. QOLIBRI is a questionnaire designed for patients recovering from brain injury and is suitable for clinical practice. It collects important information on the psychosocial elements that might be potential sources of handicap. Together, these steps should promote a biopsychosocial-spiritual model of medicine [9,10].

This project used the International Classification of Functioning, Disability and Health framework (ICF) [11]. This tool is a coding scheme that integrates biopsychosocial elements of body functions, activities, and contextual factors [11,12]. It also helps with the unifying of language between researchers. These components made the ICF suitable with the objectives of this study.

The overall goal of this retrospective study was to evaluate the post-acute coma recovery outcomes of patients having benefited from acute care in the NRA unit and to assess their needs regarding biopsychosocial elements. This should provide information on potential ways to improve clinical practice in the post-acute phase of coma recovery. This was an exploratory study aimed to guide larger potential projects in this area of research.

## 2. Materials and Methods

### 2.1. Patients

This is an exploratory case series study. Patient inclusion criteria were: (1) age ≥ 18 years; and (2) recovering from a coma and having benefited from a post-acute follow-up appointment with members of the NRA team. No exclusion criteria were applied. The first twelve patients to complete this follow-up appointment were included in our study and the follow-up took place between one and two years after injury. The first twelve post-acute follow-up appointments took place between February and May 2021. It is worthy of note that the data was collected on clinical practices taking place prior to and during the COVID-19 pandemic, and outpatient medical visits were reduced during this period. Therefore, the follow up appointments of some patients were delayed. This led to differences in the time that passed between the acute and post-acute phases. Patients having had more time to recover could therefore potentially show better outcome results.

These post-acute follow up appointments consisted of two parts. First, patients went through a neurological examination performed by a neurologist. A social and spiritual interview was then performed by a spiritual adviser. If the patient’s condition did not allow travel to the NRA unit, the follow-up interview was performed by telephone. Furthermore, if the individual was unable to communicate, the interview took place with the caregiver(s). Two out of the 12 patients had telephone interviews and three patients had interviews performed with caregivers.

Patients hospitalised in the NRA unit were classified as having clinical Cognitive Motor Dissociation (c-CMD) or Disorder of Consciousness (DoC). A “true” disorder of consciousness (DoC) is a continuum ranging from coma to Unresponsive Wakefulness Syndrome (UWS) [13] to Minimal Conscious State + (MCS+) [4,14,15]. The term CMD was introduced in 2015 [16], and describes a state in which patients have covert consciousness. In other words, their motor response fails to follow purposeful brain activation. In severe CMD cases, it can be difficult to clinically distinguish it from Unresponsive Wakefulness Syndrome (UWS). However, prognostic scales show that patients with c-CMD identified by observing their subtle motor behaviour using the MBT-r (Motor Behavior tool revised) are expected to have better long-term recovery than individuals with “true” DoC [4,17]. The NRA team identified these patients clinically using the MBT-r and confirmed the diagnosis by multimodal radiological and neurophysiological evaluation [18]. As recovery outcomes may vary significantly between these different entities [1,4,17], we included this information for the purposes of better result interpretation.

The data for this project was collected from clinical files of patients hospitalised in the NRA between December 2018 and June 2021. The information extracted included clinical scores, medical letters, and notes taken during the social and spiritual interviews.

The study was approved by Ethics Committee Vaud (CER-VD), and we obtained written informed consent from patients or their legal surrogates.

### 2.2. Outcome Assessment

#### 2.2.1. Variables Collected

The following variables were collected: age, sex, cause of brain injury, disorder of consciousness type, time between injury and post-acute follow up appointments, time between discharge, and post-acute appointments.

#### 2.2.2. Objective Outcome and Evolution

The clinical scores used in this retrospective study are those applied routinely for patient evaluation in the NRA unit. They measure mental functions, gait, and the global impact of injury on daily life.

Mental functions were assessed by the Rancho Los Amigos Level of Cognitive Functioning-Revised (LCF)-r [19] and the Disability Rating Scale (DRS) [20]. The LCF-r describes cognitive and behavioural patterns of recovery in brain injury patients. It is composed of ten levels, with Level 1 corresponding to the lowest level of function and level 10 corresponding to the highest [19]. The DRS is a functional outcome measure regarding consciousness, cognitive ability for self-care, and psychosocial adaptability [21]. It measures the impact of cognitive impairment on daily life. The minimum score is 0, corresponding to no disability, and the maximum score is 29, corresponding to an extreme vegetative state [22].

Walking functions were assessed using the Functional Ambulation Categories (FAC) [23]. It is composed of six categories, with category 0 corresponding to non-functional ambulation and category 5 to ambulatory independence.

The global impact of injury on daily life was assessed using the Glasgow Outcome Scale (GOS) [24]. This scale ranges from 1 to 5. A score of 1 corresponds to death and a score of 5 corresponds to a good recovery. The GOS was assessed using the validated French version of a structured interview introduced by Fayol et al. [25].

These scores provide an objective and global picture of the patient’s condition.

#### 2.2.3. Statistical Analysis

Clinical scores measured at discharge from the acute phase hospitalisation in the NRA unit were compared to scores measured at the follow-up appointment in the post-acute phase. Thus, for each patient, we considered scores from two points in time. We applied descriptive statistics on results from the post-acute phase and on the difference between the post-acute and acute phases, reflecting the evolution between them.

The descriptive statistics included measures of central tendency and measures of variability. These analyses were performed using IBM SPSS^®^ Statistics version 27.

#### 2.2.4. The Patient’s Point of View

We used clinical files to get information on the patients’ perspective on their conditions. These included medical letters at the time of the post-acute phase follow-up appointment as well as notes from the social and spiritual interviews performed during this visit. From these files we extracted all complaints reported by the patients. The complaints were then classified into a table form using the ICF by employing the systematic linking procedures published by Cieza et al. [26,27]. Facilitators helping patients in their recovery process were also classified using the same procedure.

Patients were asked to complete the French version of the QOLIBRI questionnaire as published by von Steinbüchel et al. [8] during the post-acute follow-up appointment. The levels of satisfaction reported in this survey were compared to related objective clinical scale results during the post-acute phase. The questionnaire was examined to identify disabilities using reference values published by Gorbunova et al. [28]. For each item of the questionnaire, a score equal to or inferior to the 16th percentile cut off value was interpreted as a complaint, as recommended in publication [28]. Reference values based on the population of The Netherlands were used, as no reference values have so far been published for the Swiss population [28]. Each complaint was then classified into an ICF category using the linking proposed by Koskinen et al. [5] (Appendix A).

The information gathered from clinical letters and QOLIBRI questionnaires was structured into a table and then presented as a diagram showing the interactions between each element.

## 3. Results

The results of this study were presented in two parts. In the first part, the neurobehavioral scores in the acute and post-acute phases were presented. In the second part, the results of patient complaints and facilitators obtained in the post-acute phase were presented.

The mean patient evolution was a Level of Cognitive Functioning Scale (LCF)-r increase of 3.33 levels, a Disability Rating Scale score (DRS) of −3.27 points, a Functional Ambulation Classification (FAC) scale score of 1.83, and the Glasgow Outcome Scale (GOS) had a median evolution of 0 points. The main patient complaints concerned mental functioning (*n* = 7), sensory functioning and pain (*n* = 6), neuromusculoskeletal and movement problems (*n* = 5), and major life areas (*n* = 5).

### 3.1. Patient Characteristics

The sample included eight males and four females. The ages ranged from 19 to 79 years. The cause of brain injury was vascular (*n* = 6) or traumatic (*n* = 6). The type of disorder of consciousness was c-CMD (*n* = 11) and “true” DoC (*n* = 1). The time between injury and the post-acute follow-up appointment ranged from 12 to 26 months. The time between discharge from acute hospitalisation in the NRA unit and the post-acute follow-up appointment ranged from 10 to 25 months.

### 3.2. Scale Results

#### 3.2.1. Mental Functions

**Figure 1:**
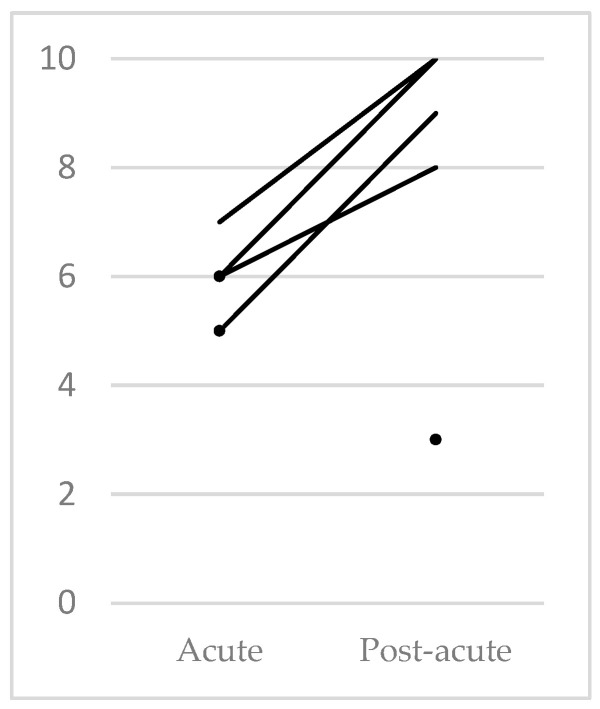
Evolution according to LCF-r. The two dots in the acute phase correspond to patients 10 and 11, who were not evaluated in the post-acute phase. The single dot present in the post-acute phase corresponds to patient 12, who was not evaluated in the acute phase.

LCF-r scores in the post-acute follow-up had a median score of 10 (interquartile range = 1.25), corresponding to cognitive level 10 (the highest score attainable). The mean evolution was 3.33 points (range = 2). However, in the post-acute phase, one patient had an LCF-r score of 3 (localised response: total assistance), and two individuals were not assessed by this method. In the acute phase, one patient was not evaluated with this scale (Table 1 and Table 2, Figure 1).

**Figure 2:**
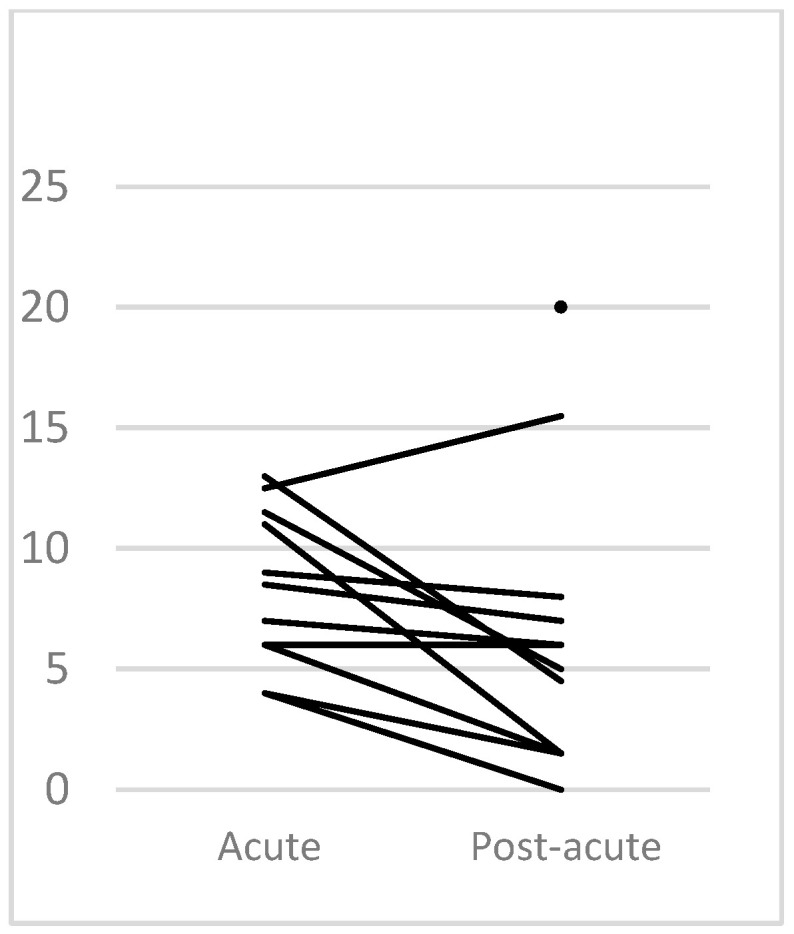
Evolution according to DRS. The dot present in the post-acute phase corresponds to patient 12, who was not evaluated in the acute phase.

The DRS showed a median score of 5.5 in the post-acute phase, corresponding to moderate disability (Interquartile range = 6.25) with a right skewed distribution (skewness = 1.39), as two patients had scores of 15.5 and 20 corresponding to severe and extremely severe disabilities, respectively. The mean evolution was −3.27 points (SD = 3.78). Of note, one patient maintained the same score and evolved unfavourably. One other patient was not evaluated by this scale in the acute stage (Table 1 and Table 2, Figure 2).

#### 3.2.2. Walking

**Figure 3:**
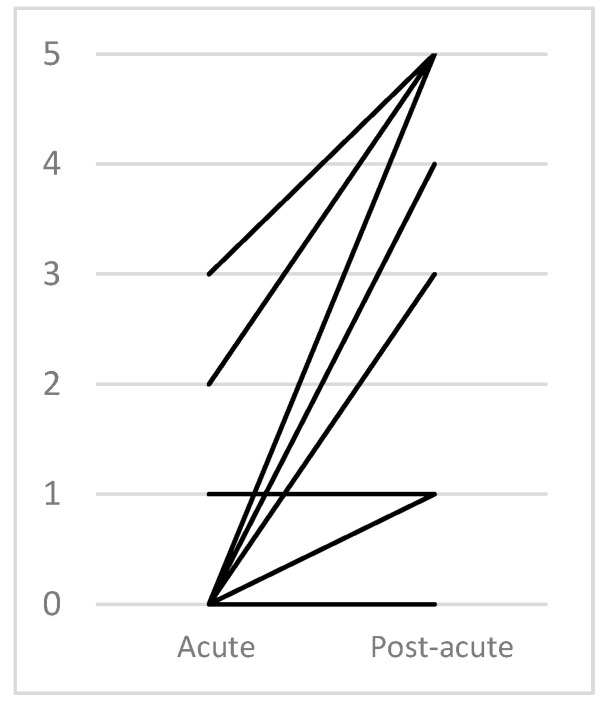
Evolution according to FAC.

The FAC showed a median score of 2.00 (interquartile range = 3.75) in the post-acute phase, with a Kurtosis = −1.71 revealing a flatter than normal distribution in our sample. Evolution had a positive mean of 1.83 (range = 5) (Table 2, Figure 3).

#### 3.2.3. Impact of Injury on Daily life

**Figure 4:**
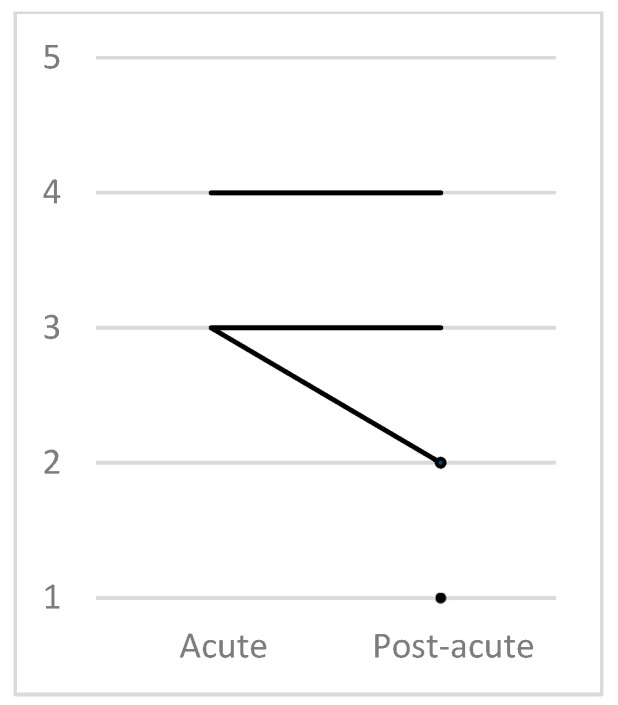
Evolution according to GOS; the dot in the post-acute phase corresponds to patient 2 and patient 5, who were not evaluated by the GOS in the acute phase.

The GOS showed a mean score of 2.58 in the post-acute phase, corresponding to severe disability (range = 3). Furthermore, we measured a median evolution of 0 (interquartile range = 1) with this scale. One patient was not evaluated in the acute phase (Table 1 and Table 2, Figure 4). The results of le GOS-Extended are detailed in Appendix D.

### 3.3. Patient Complaints

**Figure 5:**
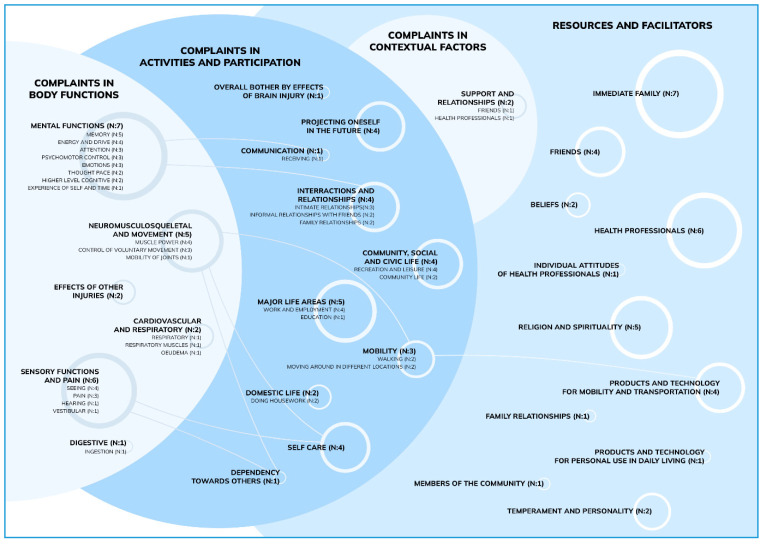
The list of complaints and resources and facilitators (Appendix B, Appendix C) of nine patients. (patient 10, patient 11 and patient 12 did not have the appropriate information). Complaints concerned body functions, activities, and participation, as well as contextual factors. We illustrated each domain as a circle, and the size of the circle relates to the number of patients (*n*) reporting that complaint. The same is true for facilitators.

In the post-acute phase, we reported complaints concerning all components of the ICF. Concerning body functions, most complaints regarded mental functions (*n* = 7), sensory functions, and pain (*n* = 6), as well as neuromusculoskeletal and movement-related functions (*n* = 5). In the activity and participation component, complaints primarily involved mobility (*n* = 3), self-care (*n* = 4), relationships (*n* = 4), major life areas (*n* = 5), community, and social and civic life (*n* = 4). We also noted two complaints in the contextual factors concerning friends (*n* = 1) and health professionals (*n* = 1) (Figure 5, Appendix C).

### 3.4. Facilitators

Notes from the social and spiritual interviews and other medical documents showed the contextual factors (for instance, the social situation or structural conditions at home) as mostly being made up of facilitators. As environmental factors (such as home conditions), we noted mainly immediate family (*n* = 7), healthcare professionals (*n* = 6), Religion and Spirituality (*n* = 5), friends (*n* = 4), products of technology for mobility, and transportation (*n* = 4) (Figure 5).

## 4. Discussion

### 4.1. Patient Characteristics

Patient ages ranged from 19 to 68 years, with causes of brain injury being either traumatic or vascular. Most subjects had a c-CMD diagnosis. The time between discharge and follow-up appointments ranged from 10–25 months.

### 4.2. Mental Functions

#### 4.2.1. Neurobehavioral Assessment Scale Results

Results of the LCF-r showed high levels of cognitive functioning in most patients. However, a level 10 on the LCF-r does not signify complete recovery. Indeed, the LCF-r mentions "modified independent", implying that some impairment may still be present. Thorough neuropsychological evaluation must be performed to have a better understanding of cognitive functioning in the post-acute phase. Nevertheless, the LCF-r results suggest that most patients attained encouraging recovery in mental functions with satisfactory cognitive and behavioural levels reached after one or two years of recovery. A previous study investigating CMD patients showed improvement in cognitive functions using LCF-r at discharge from the acute phase of hospitalisation [4], suggesting that further recovery continues into the post-acute phase.

The DRS findings evoke progress in their handicap caused by mental and cognitive disabilities in most patients, but the impact on daily life at two years of recovery is still clinically relevant. Nevertheless, in a longitudinal study, Nakase-Richardson et al. demonstrated continually improving DRS scores at five-years post injury [29], so further recovery could be expected in later phases of recovery in our patients.

Comparing the findings of DRS and LCF-r scores, we notice that the evolution in handicap measured by DRS appears to be less favourable than in mental functions measured by LCF-r from which the disabilities originated. In other words, minor measured impairment in mental functions could translate to notable limitations and restrictions in activity and participation, respectively.

#### 4.2.2. The Patient’s Point of View

At the post-acute phase follow-up, mental functioning was the source of most complaints. Difficulties primarily concerned memory, energy, drive, attention, psychomotor control, and emotions. These could be sources of difficulties concerning activities and participation. Indeed, we noted complaints in the interpersonal interactions and relationships involving informal relationships, family relationships, and intimate spousal relationships. In this same component, complaints regarding major life areas such as work and employment, community, social and civic life, and domestic life could also be partly due to impairment in mental functions.

In this respect, limitations and restrictions caused by impairments in mental function are not just objective measures but were experienced by patients in our study as sources of discomfort. This had also been reported by Koskinen et al. in a publication on rehabilitation after severe traumatic brain injury(TBI) [5].

Interestingly, we observed that a higher DRS score does not necessarily correlate with a higher number of complaints in mental function. For example, we evaluated patient 5 as having a DRS score of 1.5, corresponding to a mild level of disability. At the post-acute follow-up, this patient complained of energy and drive, attention, memory and thought pace, and organisation and planning problems. On the other hand, patient 6 and patient 8 had DRS scores of 6 and 7, respectively, corresponding to moderate and moderately severe levels of disability. However, patient 6 had no complaints regarding mental functions, and patient 8 expressed that they were only bothered in one qualifier of this component. These findings indicate that an objective evaluation of disability in mental function did not necessarily correlate with the subjective experience of handicap in our study.

In summary, we saw limitation and restriction regarding mental functions at one or two years of recovery, and objective and subjective ratings of the impact of disability were not always associated in our cohort. Overall, the satisfaction rate of mental function was rather optimistic, as seen from the QOLIBRI questionnaire indicating self-reported satisfaction levels of 71.36% and 82.22% on average for the cognition and emotion scales, respectively.

### 4.3. Ambulation

#### 4.3.1. Ambulatory Scale Results

FAC results showed very little uniformity. Some patients showed very good outcomes in gait, with several evolving from non-functional ambulation to independent ambulation. However, others showed very little or no progress at all. As mentioned by Dever et al., brain injuries differ between each patient, and the impact on specific regions involved in gait may vary among individuals [30]. Furthermore, our small sample size may be a cause of disparity in our data [31].

#### 4.3.2. The Patient’s Point of View

At the post-acute follow-up, mobility and walking were a source of complaint for only three patients (patient 1, patient 4, and patient 9). Interestingly, we note that patient 1 and patient 9 had FAC scores of 4 and 3, respectively, (level 3 corresponds to walking on a level surface with supervision; level 4 corresponds to independent walking on level surfaces) which are among the highest scores of our sample, indicating that an objective evaluation of gait did not necessarily reflect the level of discomfort an individual may experience by his limitation in this activity in our patients.

Furthermore, in the QOLIBRI questionnaire, this component had an average self-reported satisfaction rate of 61.11%, leading us to suppose that even if our patients do not necessarily complain about their limitation in walking, they are not very satisfied by the situation.

In the post-acute phase, therefore, we did not find consistency in the level of ambulation or satisfaction in our sample. As with mental functions, the objective scoring of gait does not necessarily correlate with the patient’s expressed discomfort in our study.

### 4.4. The Impact of Injury on Daily Life

#### 4.4.1. GOS Scale Results

Results of the GOS score suggest a high handicap persisting in most patients after one or two years of recovery, and we saw very little evolution in this score. This indicates that levels of independence for daily activities evaluated at discharge from the unit remained relatively constant for the two following years of recovery in our cohort. We also noted that none of our 12 patients had returned to work by the post-acute follow-up.

Similar results were found in other studies evaluating the chronic effect of injury on the daily life of patients with severe TBI [32,33]. Furthermore, the functional outcome of patients with CMD at one year of injury was also investigated by Claassen et al. using the GOSE [17]. In their publication, 7 out of 16 patients with CMD had an upper severe disability score of 4/8 or better. This score is similar to the findings of our study.

#### 4.4.2. The Patient’s Point of View

The impact of injury on daily life was a frequent source of complaint by patients at the post-acute phase follow-up. It covered self-care, domestic life, interactions and relationships, work, employment, and education, as well as community and social and civic life (*n* = 4).

In a study by Koskinen et al., they also noted that employment was a major source of disappointment amongst patients recovering from severe TBI [5].

Regarding daily life and autonomy, the QOLIBRI questionnaire indicated an average reported satisfaction rate of 60.24%. Compared to the results of the GOS scores, we consider this level of satisfaction to be relatively optimistic.

In conclusion, in the post-acute phase, a significant handicap was still present for daily life activities. This was measured using GOS and was a frequent source of complaint in our patients.

### 4.5. DOC and CMD

The patient diagnosed as DOC in the acute stage had lower scores in the post-acute phase than patients with a c-CMD diagnostic. On scales assessing mental functions, the patient had a score of 3 on the LCF-r, corresponding to localized response: total assistance, and a DRS score of 20, corresponding to extremely severe disability. Furthermore, the GOS score of this patient was 4, corresponding to a persisting vegetative state. This finding is in line with the observed better recovery in patients diagnosed with CMD [17,34], although we are considering one patient only.

Due to this patient’s condition, we could not perform the QOLIBRI questionnaire and the social and spiritual interview in the post-acute follow-up appointment and have no subjective information on the experience of the situation. There was therefore a risk of interpreting his condition by solely relying on scale results, which we believe would be fundamentally insufficient.

Additional and broader studies need to be performed to compare the long-term evolution of c-CMD and DOC in more depth.

### 4.6. Primary Complaints

In the post-acute phase, most complaints regarded mental functions, sensory functions, and pain, as well as neuromusculoskeletal and movement-related functions. These impairments, often as combinations, could explain complaints in activities and participation such as mobility, self-care, relationships, major life areas, community, and social and civic life.

These biological, psychological, and social difficulties indicate a necessity for a holistic patient management approach in coma recovery. This requires clinicians to be aware of the broader implications that impairments can have on individual patient activities, participation, and environment, although our findings need to be supported in a larger study [7].

#### Individuality

Interestingly, there is no single element listed as a complaint by all patients. Furthermore, some patients expressed difficulties in more components than others did. Therefore, one specific type of outcome in the post-acute phase cannot be generalised for all individuals recovering from coma in our study. The brain injury types and locations affecting specific body functions vary for each person. Moreover, each patient evolves along their own path, which can facilitate (or not) the activities and participation they engage in. Thus, a personalized approach should be encouraged.

### 4.7. Facilitators

Notes from the social and spiritual interviews and other medical documents showed contextual factors (for instance good family support) as being mostly facilitators.

As mentioned in several studies on rehabilitation after TBI [6,7,35], family is often an important resource for patients. Furthermore, most facilitators reported in this study concerned human interaction. This emphasizes the importance of the social environment of each patient in our study and a potential need for clinicians to understand how to integrate it into patient management on an individual basis.

Limitations in interactions and relationships were a recurrent source of complaint in this case series (*n* = 4). This concerned informal relationships, family relationships, and intimate relationships. Other authors reported similar findings during recovery after TBI [1,32,36].

Patients found resources in domains classified according to ICF as activity and participation. For example, religion and spirituality were frequently observed as sources of support (42%) in our cohort. This finding highlights a potential opportunity for hospital staff to discuss this aspect with patients and to offer spiritual counselling, as it may play an important role in a patient’s rehabilitation process if observed in larger cohorts.

Two patients mentioned contextual factors as being barriers. These involved friends and health professionals. This may indicate that if the identified facilitators do not continue to support the patient, they might become obstacles in their recovery process. It might be useful to investigate the role that contextual factors play for individuals.

### 4.8. Differences between Clinical Scale Results and a Patient’s Point of View

As several authors previously stated regarding outcome measurements in TBI [1,33], the objective evaluation of restrictions and limitations by clinical scores does not always correlate with the subjective level of discomfort patients describe for their handicap. The results of our study indicated this regarding the FAC and DRS scales. We try to explain this divergence with the following contextual factors.

Patient 8 and patient 9 had many similarities. Both had suffered a traumatic brain injury, both had FAC scores of 1 in the post-acute phase, and other than walking, they expressed similar complaints. In addition, they had contextual factor facilitators ranging from technical aid for walking and professional health assistance to family support and spiritual life. Nonetheless, they differed greatly in their level of satisfaction towards their walking ability. There was a personal factor mentioned in the social and spiritual interview for patient 8, the patient’s personality as a facilitator, but this was not mentioned by patient 9. We can hypothesise that for patient 8 this led to a better coping mechanism when facing walking difficulties as compared to patient 9.

A limitation of this study is the retrospective methodology, making it difficult to explore the importance that contextual factors have on each patient and their specific limitations more deeply. Nonetheless, because of similarities in the qualifiers, their meaning and effect may be expected to be different for each patient.

Dikmen et al. suggested that, depending on the severity of the injury, patients might present a lack of awareness of their problems or fail to appreciate their significance. They also stated that concerns about functioning might be outweighed by the appreciation of survival [32].

In the same way, Gasquoine mentions that subjective information, such as levels of satisfaction, could be altered by anosognosia and other forms of neglect resulting from brain injury [37]. Depending on the severity of the conditions, self-reported data could underestimate the importance of a handicap. Detailed neuropsychological evaluations studied simultaneously could enhance the interpretation.

In conclusion, we suggest the importance of exploring individual contextual factors such as environmental factors, psychological functioning, and spiritual beliefs that could be barriers or facilitators of one’s experience of one’s handicap.

Most current work in the literature used purely objective outcome measurements in the follow-up phase [17,29]. Some studies reported on patient viewpoints but focused on TBI, not on disorders of consciousness [5,32,35,36]. This signifies the need for more research on the post-acute outcomes of patients recovering from coma.

### 4.9. Authors’ Suggestions

The following remarks are intended to extend the conversation surrounding biopsychosocial and spiritual management when considering the management of patients with severe brain lesions. These comments and suggestions are based on our current experience in the NRA unit and could be a starting point for further research.

First, we noted that during the post-acute follow up visits, patients and their families often have many unanswered questions regarding events that took place in the acute phase and for their current situation. Although this can be time consuming, we believe it is important for clinicians to take the time to discuss questions, as they can be a source of misunderstanding and a great burden for patients.

We further noted that having a post-acute follow up visit in the same unit in which the patient had been hospitalised during the acute phase of his recovery seemed to be of high importance. Indeed, we regularly hear from patients and families that it helps them bring some closure to a difficult part of their journey.

Moreover, when discussing social and spiritual components with patients, we realised that spiritual advisors are often more skilled than physicians at investigating these elements and in understanding the way they affect the patient. On the contrary, in such interviews, the importance of the doctor-patient relationship should not be underestimated. Indeed, the faith and trust that patients have in their doctor is often crucial for deeper conversations leading to a better understanding of patient needs. Thus, when investigating social and spiritual elements, we recommend performing the interview in the presence of the physician and a spiritual counsellor.

Regarding severe brain damage management, a significant amount of resources are necessary for acute care. We believe that this investment should be better correlated with patient outcome. A patient’s rehabilitation path should, in our opinion, be tailored individually according to specific patient needs. Finally, professional and social reinsertion should be given more attention.

### 4.10. Limitations

The main limitations of this study were the sample size and the retrospective methodology. The small number of participants made it difficult to generalize the findings to a wider population. The retrospective method limited our ability to investigate the subjective information such as complaints or individual effects of contextual factors in a deeper way. However, our study could serve as a starting point for future larger studies.

Furthermore, the wide eligibility criteria could make the data difficult to interpret [31]. However, our study was exploratory. This heterogeneity reflected the reality of the clinical setting, where patients are individuals with variable characteristics.

In addition, subjective information such as quality of life may be altered by anosognosia and other forms of neglect resulting from brain injury [37]. Detailed neuropsychological evaluations studied simultaneously might enhance the interpretation of the self-reported data.

The data collection method based on clinical files is also a limitation and is a source of missing or incomplete information.

## 5. Conclusions

In the post-acute phase of recovery, most patients included in this study, even those with minor impairment in mental functions, expressed important limitations. Mental functions were also the main source of complaint at the post-acute follow-up. Concerning walking, we found heterogeneity in outcomes, with some patients having completely recovered and others for whom we observed limited progress. Regarding the impact of brain injury on daily life, we noted that most patients still had a significant handicap. Patient complaints in this phase of recovery indicated difficulties in all parts and components of the ICF. This demonstrates a global burden involving biopsychosocial and spiritual elements interacting with one another. Furthermore, we observed that objective evaluation using clinical scoring does not necessarily correlate with the subjective representation patients have of their condition. We also report the individuality of each situation, with disparities between patients in terms of impairment in body functions, the effect on activities, and the relevant contextual factors affecting them. These findings emphasize a need for a personal and holistic approach to coma recovery management in the post-acute setting that is assessed in large multi-centre clinical studies.

## Figures and Tables

**Table 1:** Clinical scale results.

SCALE		LCF-r		DRS		FAC		GOS	
Patient	Disorder Type	Acute	Post-Acute	Acute	Post-Acute	Acute	Post-Acute	Acute	Post-Acute
1	c-CMD	6	10	11	1.5	0	4	3	2
2	c-CMD	7	10	4	0	2	5	-	1
3	c-CMD	6	10	6	1.5	0	5	3	2
4	c-CMD	7	10	6	6	1	1	3	3
5	c-CMD	7	10	4	1.5	3	5	-	2
6	c-CMD	7	10	7	6	0	1	3	3
7	c-CMD	5	9	13	4.5	0	3	3	2
8	c-CMD	6	8	8.5	7	1	1	3	3
9	c-CMD	5	9	11.5	5	0	3	3	3
10	c-CMD	5	-	12.5	15.5	0	1	3	3
11	c-CMD	6	-	9	8	0	0	3	3
12	DOC	-	3	-	20	0	0	4	4

**Table 2:** Scale statistics.

LCF-r		DRS		FAC		GOS	
Post-acute phase							
Median:	10	Median	5.50	Median	2.00	Mean	2.58
Interquartile Range	1.25	Interquartile Range	6.25	Interquartile Range	3.75	Range	3.00
Evolution (Post-acute—Acute)							
Mean:	3.33	Mean	−3.27	Mean	1.83	Mean	0.00
Range:	2.00	SD	3.78	Range	5.00	Interquartile range	1.00

## Data Availability

The authors confirm that the data supporting the findings of this study are available within the article.

## Appendix A

### Methodology of QOLIBRI Results

The answers to the QOLIBRI questionnaires are presented in Table A1. The age and gender of patients are presented in Table A2. For each patient, with respect to age, gender, and health status, we compared the answers to the reference values (NL) published by Gorbunova et al. [28], where the authors suggest a cut-off value of 16%. Values under 16% indicate impaired Health Related Quality of Life (HRQoL). We considered such self-reported impairment in HRQoL as a complaint and classified the corresponding category in our table of complaints (Appendix B).

**Table A1:** Patient answers to QOLIBRI.

A: Satisfaction in thinking abilities									
Patient	A.1 (b140)	A.2 (d310/d330/d350)	A.3 (b144)	A.4 (d175)	A.5 (b164)	A.6 (b1565)	A.7 (b160)	mean	mean [%]
01	3	5	4	4	4	5	5	4.29	82.14
02	4	4	5	4	4	5	4	4.29	82.14
03	4	4	3	-	5	3	3	3.67	66.67
04	5	5	3	5	5	5	4	4.57	89.29
05	3	2	2	3	3	3	2	2.57	39.29
06	5	5	4	4	5	5	5	4.71	92.86
07	3	5	1	-	2	4	4	3.17	54.17
08	3	3	5	-	3	5	5	4	75
09	2	5	2	5	4	3	3	3.43	60.71
B: Satisfaction in emotions									
Patient	B.1 (b130)	B.2 (b1301)	B.3 (b180)	B.4 (b1801)	B.5 (nd)	B.6 (b180)	B.7 (nd)	mean	mean [%]
01	4	4	3	4	4	3	4	3.71	67.86
02	4	4	4	4	4	4	4	4.00	75.00
03	4	4	5	5	5	5	4	4.57	89.29
04	2	3	4	4	4	4	3	3.43	60.71
05	2	3	3	3	3	3	3	2.86	46.43
06	5	5	5	5	5	5	3	4.71	92.86
07	5	5	2	5	5	4	1	3.86	71.43
08	3	3	3	3	5	3	3	3.29	57.14
09	5	4	4	4	5	4	1	3.86	71.43
C: Satisfaction in daily life									
Patient	C.1 (d5)	C.2 (d460)	C.3 (d640)	C.4 (d860)	C.5 (d850/d820)	C.6 (d910/d920)	C.7 (nd)	mean	mean [%]
01	3	3	4	-	2	4	4	3.33	58.33
02	5	5	5	5	5	5	5	5.00	100.00
03	4	5	5	4	-	5	4	4.50	87.50
04	3	3	2	-	3	3	4	3.00	50.00
05	2	3	4	3	3	2	3	2.86	46.43
06	2	3	3	3	4	5	4	3.43	60.71
07	4	4	3	-	-	3	3	3.40	60.00
08	1	4	3	-	3	4	3	3.00	50.00
09	3	1	1	-	1	4	3	2.17	29.17
D: Satisfaction in relations									
	Patient	D.1 (b152)	D.2 (d760)	D.3 (d750)	D.4 (d7701)	D.5 (d7702)	D.6 (e4)	mean	mean [%]
	01	5	5	4	5	5	5	4.83	95.83
	02	5	5	5	5	5	4	4.83	95.83
	03	5	5	5	5	5	5	5.00	100.00
	04	5	5	5	5	-	5	5.00	100.00
	05	4	4	3	4	4	4	3.83	70.83
	06	5	5	5	3	1	5	4.00	75.00
	07	4	5	4	5	3	4	4.17	79.17
	08	3	5	2	5	1	5	3.50	62.50
	09	4	4	2	1	1	4	2.67	41.67
E: Bothered by feelings									
		Patient	E.1 (b152)	E.2 (b152)	E.3 (b152)	E.4 (b152)	E.5 (b152)	mean	mean [%]
		01	5	3	5	4	3	4	75
		02	5	4	5	5	5	4.8	95
		03	5	5	5	5	5	5	100
		04	5	5	5	5	5	5	100
		05	3	5	5	4	2	3.8	70
		06	5	5	5	5	5	5	100
		07	4	4	4	5	2	3.8	70
		08	5	5	3	1	5	3.8	70
		09	4	3	3	3	4	3.4	60
F: Bothered by physical problems									
		Patient	F.1 (b1470/b760)	F.2 (nd)	F.3 (b280)	F.4 (b210/b230)	F.5 (nd)	mean	mean [%]
		01	1	5	5	5	3	3.80	70.00
		02	5	3	5	5	4	4.40	85.00
		03	2	5	5	4	5	4.20	80.00
		04	1	1	5	1		2.00	25.00
		05	3	1	4	2	4	2.80	45.00
		06	5	3	5	3	3	3.80	70.00
		07	3	-	3	5	2	3.25	56.25
		08	5	5	5	3	3	4.20	80.00
		09	5	5	1	5	2	3.60	65.00

The parentheses represent the ICF domains to which the QOLIBRI question corresponds. Each patient rates a satisfaction level or burden on a scale from 1 to 5.

**Table A2:** Patient age and gender.

Patient	Age	Gender
Patient 1	50	F
Patient 2	33	M
Patient 3	23	M
Patient 4	68	M
Patient 5	26	M
Patient 6	53	F
Patient 7	54	M
Patient 8	41	M
Patient 9	29	F
Patient 10	79	F
Patient 11	59	M
Patient 12	19	M

## Appendix B

**Table A3:** List and categorisation of complaints according to ICF.

Patient	Component	Constructs		Domain	ICF Code		Source
PATIENT01	Body Functions	B1-Mental Functions	Global	Energy and Drive-Energy Level-	B130	0	Med. let
PATIENT01	Body Functions	B1-Mental Functions	Specific	Attention	B140	0	QOLIBRI + Med. let.
PATIENT01	Body Functions	B1-Mental Functions	Specific	Psychomotor-Control	B147	0	QOLIBRI
PATIENT01	Body Functions	B7-Neuromusculoskeletal and Movement	Muscles	Muscle Power	b730	2	Med. let
PATIENT01	Body Functions	B7-Neuromusculoskeletal and Movement	Movement	Control of voluntary Movement	B760		QOLIBRI
PATIENT01	Activities & Participation	D4-Mobility	Walking and moving	Walking	D450		Med. let.
PATIENT01	Activities & Participation	D8-Major Life Areas	Work and Employment	Remunerative Employment	D850		QOLIBRI + Med. let. + interview
PATIENT02	Body Functions	B2-Sensory Functions and Pain	Hearing and Vestibular	Vestibular	B235	1	Med. let.
PATIENT03	Body Functions	B1-Mental Functions	Specific	Memory	B144		Med. let.
PATIENT03	Body Functions	B1-Mental Functions	Specific	Psychomotor-Control	B147	0	QOLIBRI + Med. let.
PATIENT03	Body Functions	B1-Mental Functions	Specific	Thought pace	B160	0	Med. let.
PATIENT03	Body Functions	B7-Neuromusculoskeletal and Movement	Muscles	Muscle Power	B730		Med. let.
PATIENT03	Body Functions	B7-Neuromusculoskeletal and Movement	Movement	Control of voluntary Movement	B760		QOLIBRI
PATIENT03	Activities & Participation	D8-Major Life Areas	Work and Employment	Acquiring, Keeping and Terminating a Job	D845		Med. let.
PATIENT03	Environmental Factors	E3-Support and Relationships		Health Professionals	E355		Med. let.
PATIENT04	Body Functions	B1-Mental Functions	Global	Energy and Drive-Motivation	B130	1	QOLIBRI
PATIENT04	Body Functions	B1-Mental Functions	Global	Energy and Drive-Energy Level-	B130	0	QOLIBRI
PATIENT04	Body Functions	B1-Mental Functions	Specific	Memory	B144		QOLIBRI
PATIENT04	Body Functions	B1-Mental Functions	Specific	Psychomotor-Control	B147	0	QOLIBRI
PATIENT04	Body Functions	B2-Sensory Functions and Pain		Seeing	B210		QOLIBRI + Med. let.
PATIENT04	Body Functions	B2-Sensory Functions and Pain	Hearing and Vestibular	Hearing	B230		QOLIBRI
PATIENT04	Body Functions	B4-Cardiovascular, Haematological, Immunological and Respiratory	Respiratory	Respiration	B440		Med. let.
PATIENT04	Body Functions	B4-Cardiovascular, Haematological, Immunological and Respiratory	Respiratory	Respiratory Muscles	B445		Med. let.
PATIENT04	Body Functions	B5-Digestive, Metabolic and Endocrine	Digestive	Ingestion-Swallowing	B510	5	Med. let.
PATIENT04	Body Functions	B5-Digestive, Metabolic and Endocrine	Digestive	Ingestion-Salivation	B510	4	Med. let.
PATIENT04	Body Functions	B7-Neuromusculoskeletal and Movement	Muscles	Muscle Power	B730	2	Med. let.
PATIENT04	Body Functions	B7-Neuromusculoskeletal and Movement	Movement	Control of voluntary Movement	B760		QOLIBRI
PATIENT04	Activities & Participation	D4-Mobility	Walking and moving	Walking	D450		Med. let.
PATIENT04	Activities & Participation	D4-Mobility	Walking and moving	Moving around in different locations	D460		QOLIBRI
PATIENT04	Activities & Participation	D6-Domestic Life	Household tasks	Doing Housework	D640		QOLIBRI
PATIENT04	Activities & Participation	D7-Interpersonnal Interactions and Relationships	Particular interpersonal relationships	Family relationships- parent-child	D760		interview
PATIENT04	Activities & Participation	D8-Major Life Areas	Work and Employment	Remunerative Employment	D850		QOLIBRI
PATIENT04	Activities & Participation	D9-Community, Social & Civic Life		Community Life	D910		QOLIBRI
PATIENT04	Activities & Participation	D9-Community, Social & Civic Life		Recreation and leisure	D920		QOLIBRI
PATIENT04	Activities & Participation	D5-Self Care	Self-care				QOLIBRI
PATIENT04	nd			Projecting oneself into the future			QOLIBRI
PATIENT04	nd-gh			Other injuries sustained at time of brain injury			QOLIBRI
PATIENT05	Body Functions	B1-Mental Functions	Global	Energy and Drive	B130	0	QOLIBRI + Med. let.
PATIENT05	Body Functions	B1-Mental Functions	Specific	Attention	B140	0	Med. let.
PATIENT05	Body Functions	B1-Mental Functions	Specific	Memory	B144		QOLIBRI
PATIENT05	Body Functions	B1-Mental Functions	Specific	Thought pace	B160	0	QOLIBRI + Med. let.
PATIENT05	Body Functions	B1-Mental Functions	Specific	Higher-level Cognitive- Organization and Planning	B164	1	Med. let.
PATIENT05	Activities & Participation	D3-Communication	Receiving	Receiving-Spoken	D310		QOLIBRI
PATIENT05	Activities & Participation	D8-Major Life Areas	Work and Employment	Acquiring, Keeping and Terminating a Job	D845		Med. let.
PATIENT05	Activities & Participation	D9-Community, Social & Civic Life		Community Life	D910		QOLIBRI
PATIENT05	Activities & Participation	D9-Community, Social & Civic Life		Recreation and leisure	D920		QOLIBRI + Med. let. + interview
PATIENT05	Activities & Participation	D5-Self Care	Self-Care				QOLIBRI
PATIENT05	nd			Projecting oneself into the future			Med. let.
PATIENT05	nd-gh			Other injuries sustained at time of brain injury			QOLIBRI
PATIENT06	Body Functions	B2-Sensory Functions and Pain	Seeing and related	Seeing	B210		Med. let.
PATIENT06	Body Functions	B2-Sensory Functions and Pain	Pain	Sensation of Pain	B280		Med. let.
PATIENT06	Body Functions	B7-Neuromusculoskeletal and Movement	Muscles	Muscle Power	B730	2	Med. let.
PATIENT06	Activities & Participation	D7-Interpersonnal Interactions and Relationships	Particular interpersonal relationships	Intimate relationships-sexual	D770	2	QOLIBRI
PATIENT06	Activities & Participation	D5-Self Care	Self-Care				QOLIBRI
PATIENT06	Body Functions	B4-Cardiovascular, Haematological, Immunological and Respiratory	Unspecified	Unspecified	B429		Med. let.
PATIENT07	Body Functions	B1-Mental Functions	Global	Energy and Drive	B130	0	Med. let.
PATIENT07	Body Functions	B1-Mental Functions	Specific	Memory	B144		QOLIBRI + Med. let.
PATIENT07	Body Functions	B1-Mental Functions	Specific	Emotions	B152		QOLIBRI
PATIENT07	Body Functions	B1-Mental Functions	Specific	Higher-level Cognitive	B164		QOLIBRI
PATIENT07	Body Functions	B1-Mental Functions	Specific	Experience of Self and Time	B180		QOLIBRI
PATIENT07	Body Functions	B2-Sensory Functions and Pain	Pain	Sensation of Pain	B280		Med. let.
PATIENT07	Activities & Participation	D9-Community, Social & Civic Life		Recreation and leisure	D920		Med. let.
PATIENT07	nd			Projecting oneself into the future			QOLIBRI
PATIENT08	Body Functions	B1-Mental Functions	Specific	Affect	B152		QOLIBRI + Med. let.
PATIENT08	Body Functions	B2-Sensory Functions and Pain	Seeing and related	Seeing	B210		Med. let.
PATIENT08	Activities & Participation	D7-Interpersonnal Interactions and Relationships	Particular interpersonal relationships	Informal Relationships-with Friends	D750	0	QOLIBRI + interview
PATIENT08	Activities & Participation	D7-Interpersonnal Interactions and Relationships	Particular interpersonal relationships	Family relationships	D760	0	interview
PATIENT08	Activities & Participation	D7-Interpersonnal Interactions and Relationships	Particular interpersonal relationships	Intimate relationships-sexual	D770	2	QOLIBRI
PATIENT08	Activities & Participation	D9-Community, Social & Civic Life		Recreation and leisure	D920		Med. let.
PATIENT08	Activities & Participation	D5-Self Care	Self-care				QOLIBRI
PATIENT08	Environmental Factors	E3-Support and Relationships		Friends	E320		interview
PATIENT08	nd			Dependency towards others			Med. let. + interview
PATIENT09	Body Functions	B1-Mental Functions	Specific	Attention	B140		QOLIBRI
PATIENT09	Body Functions	B1-Mental Functions	Specific	Memory	B144		QOLIBRI + Med. let.
PATIENT09	Body Functions	B1-Mental Functions	Specific	Anxiety	B152		Med. let.
PATIENT09	Body Functions	B1-Mental Functions	Specific	Affect	B152		Med. let.
PATIENT09	Body Functions	B2-Sensory Functions and Pain	Seeing and related	Seeing	B210		Med. let.
PATIENT09	Body Functions	B2-Sensory Functions and Pain	Pain	Sensation of Pain	B280	10	QOLIBRI + Med. let.
PATIENT09	Body Functions	B7-Neuromusculoskeletal and Movement	Joints & Bones	Mobility of Joints	B710		Med. let.
PATIENT09	Activities & Participation	D4-Mobility	Walking and moving	Moving around in different locations	D460		QOLIBRI
PATIENT09	Activities & Participation	D6-Domestic Life	Household tasks	Doing Housework	D640		QOLIBRI
PATIENT09	Activities & Participation	D7-Interpersonnal Interactions and Relationships	Particular interpersonal relationships	Informal Relationships-with Friends	D750	0	QOLIBRI + med. let.
PATIENT09	Activities & Participation	D7-Interpersonnal Interactions and Relationships	Particular interpersonal relationships	Intimate relationships-spousal	D770	1	QOLIBRI
PATIENT09	Activities & Participation	D7-Interpersonnal Interactions and Relationships	Particular interpersonal relationships	Intimate relationships-sexual	D770	2	QOLIBRI
PATIENT09	Activities & Participation	D8-Major Life Areas	Education	Higher Education	D830		QOLIBRI
PATIENT09	nd			Overall bother by effects of brain injury			QOLIBRI
PATIENT09	nd			Projecting oneself into the future			QOLIBRI + med. let.

Table A3 lists the complaints and their ICF code. The “source” column corresponds to the location where the information was obtained. Med let. (Medical letter), QOLIBRI, Interview (notes from the spiritual adviser’s interview).

**Table A4:** List and categorization of resources and facilitators.

Patient	Component	Domain	ICF Code	SOURCE
PATIENT01	Environmental factors	Friends	E320	interview
PATIENT01	Environmental factors	Immediate family	E310	interview
PATIENT01	Personal factors	Beliefs		interview
PATIENT03	Environmental factors	Friends	E320	interview
PATIENT03	Environmental factors	Health Professionals	E355	Med. let.
PATIENT03	Environmental factors	Immediate family	E310	Med. let. + interview
PATIENT03	Environmental factors	Products and technology for personal use in daily living	E115	Med. let.
PATIENT04	Environmental factors	Health Professionals	E355	
PATIENT04	Environmental factors	Immediate family	E310	
PATIENT04	Environmental factors	Products and technology for mobility and transportation	E120	
PATIENT05	Activities & Participation	Religion and spirituality	D930	interview
PATIENT05	Environmental factors	Friends	E320	interview
PATIENT05	Environmental factors	Immediate family	E310	Med. let. + interview
PATIENT05	Environmental factors	Individual attitudes of health professionals	E450	interview
PATIENT05	Personal factors	Beliefs		interview
PATIENT06	Activities & Participation	Family relationships	D760	Med. let. + interview
PATIENT06	Activities & Participation	Religion and spirituality	D930	interview
PATIENT06	Body Functions	Temperament and personality functions	B126	interview
PATIENT06	Environmental factors	Friends	E320	interview
PATIENT06	Environmental factors	Health Professionals	E355	Med. let.
PATIENT06	Environmental factors	Products and technology for mobility and transportation	E120	Med. let.
PATIENT07	Activities & Participation	Religion and spirituality	D930	interview
PATIENT07	Environmental factors	Health Professionals	E355	Med. let.
PATIENT07	Environmental factors	Immediate family	E310	Med. let. + interview
PATIENT08	Activities & Participation	Religion and spirituality	D930	interview
PATIENT08	Body Functions	Temperament and personality functions	B126	Med. let. + interview
PATIENT08	Environmental factors	Health Professionals	E355	Med. let.
PATIENT08	Environmental factors	Immediate family	E310	Med. let. + interview
PATIENT08	Environmental factors	Products and technology for mobility and transportation	E120	Med. let.
PATIENT09	Activities & Participation	Religion and spirituality	D930	interview
PATIENT09	Environmental factors	Acquaintances, peers, colleagues, neighbours and community members	E325	interview
PATIENT09	Environmental factors	Health Professionals	E355	Med. let.
PATIENT09	Environmental factors	Immediate family	E310	Med. let. + interview
PATIENT09	Environmental factors	Products and technology for mobility and transportation	E120	Med. let.

## Appendix C

**Table A5:** Number of patients per complaint with ICF categorization.

Body functions	**B1-Mental Functions (*n* = 7)**			**B2-Sensory Functions and Pain (*n* = 6)**			**B4-Cardiovascular and Respiratory (*n* = 2)**			**B5-Digestive (*n* = 1)**		**B7-Neuromusculoskeletal and Movement (*n* = 5)**		
	Domain		*n*	Domain	*n*		Domain		*n*	Domain	*n*	Domain		*n*
	B144-Memory		5	B210-Seeing	4		B440-Respiratory		1	Ingestion	1	B730-Muscle Power		4
	B130-Energy and Drive		4	B280-Sensation of Pain	3		B445-Respiratory Muscles		1			B760-Control of voluntary Movement		3
	B140-Attention		3	B230-Hearing	1		B429-Oedema		1			B710-Mobility of Joints		1
	B147-Psychomotor-Control		3	B235-Vestibular	1									
	B152-Emotional Functions		3											
	B160-Thought-pace		2											
	B164-Higher-level Cognitive		2											
	B180-Experience of Self and Time		1											
Activity and Participation	**D3-Communication (*n* = 1)**		**D4-Mobility (*n* = 3)**		**D5-Self Care (*n* = 4)**		**D6-Domestic Life (*n* = 2)**		**D7-Interpersonnal Interractions and Relationships (*n* = 4)**		**D8-Major Life Areas (*n* = 5)**		**D9-Community, Social & Civic Life (*n* = 4)**	
	Domain	*n*	Domain	*n*	Domain	*n*	Domain	*n*	Domain	*n*	Domain	*n*	Domain	*n*
	Receiving-Spoken	1	Walking	2	nd	4	Doing House-work	2	Intimate relationships	3	Work and Employ-ment	4	Commun-ity Life	2
			Moving around in different locations	2					Informal Relationships-with Friends	2	Education	1	Recrea-tion and leisure	4
									Family relationships	2				
Environmental factors	**E3-Support and Relationships (*n* = 2)**													
	Friends												1	
	**Health Professionals**												1	
**nd**	Nd (*n* = 5)													
	Projecting oneself into the future												4	
	Other injuries sustained at time of brain injury												2	
	Dependency towards others												1	
	Overall bother by effects of brain injury												1	

Table A5 orders the information from Table A3 (Appendix B) according to the number of patients presenting a complaint in each category.

**Table A6:** Number of patients per resource and facilitator.

Domain	*n*
Immediate family	7
Health Professionals	6
Religion and spirituality	5
Products and technology for mobility and transportation	4
Friends	4
Beliefs	2
Temperament and personality functions	2
Individual attitudes of health professionals	1
Acquaintances, peers colleagues, neighbours and community members	1
Products and technology for personal use in daily living	1
Family relationships	1

Table A6 orders the information of Table A4 (Appendix B) according to the number of patients presenting a complaint in each category.

## Appendix D

**Table A7:** GOSE results.

GOSE Score	*n*
4 Vegetative state	1
3.2 Lower severe disability	4
3.1 Upper severe disability	2
2.2 Lower moderate disability	3
2.1 Upper moderate disability	1
1.2 Lower good recovery	0
1.1 Upper good recovery	1

## References

[1] Pignat J.-M., Mauron E., Jöhr J., de Keranflec’H C.G., Van De Ville D., Preti M.G., Meskaldji D.E., Hömberg V., Laureys S., Draganski B. (2016). Outcome Prediction of Consciousness Disorders in the Acute Stage Based on a Complementary Motor Behavioural Tool. PLoS ONE.

[2] Edlow B.L., Chatelle C., Spencer C.A., Chu C.J., Bodien Y.G., O’connor K.L., Hirschberg R.E., Hochberg L.R., Giacino J.T., Rosenthal E.S. (2017). Early detection of consciousness in patients with acute severe traumatic brain injury. Brain.

[3] Pincherle A., Rossi F., Jöhr J., Dunet V., Ryvlin P., Oddo M., Schiff N., Diserens K. (2020). Early discrimination of cognitive motor dissociation from disorders of consciousness: Pitfalls and clues. J. Neurol..

[4] Jöhr J., Halimi F., Pasquier J., Pincherle A., Schiff N., Diserens K. (2020). Recovery in cognitive motor dissociation after severe brain injury: A cohort study. PLoS ONE.

[5] Koskinen S., Hokkinen E.-M., Wilson L., Sarajuuri J., Von Steinbüchel N., Truelle J.-L. (2011). Comparison of subjective and objective assessments of outcome after traumatic brain injury using the International Classification of Functioning, Disability and Health (ICF). Disabil. Rehabil..

[6] Borgen I.M.H., Løvstad M., Andelic N., Hauger S., Sigurdardottir S., Søberg H.L., Sveen U., Forslund M.V., Kleffelgård I., Lindstad M. (2020). Traumatic brain injury—needs and treatment options in the chronic phase: Study protocol for a randomized controlled community-based intervention. Trials.

[7] Wright C.J., Zeeman H., Biezaitis V. (2016). Holistic Practice in Traumatic Brain Injury Rehabilitation: Perspectives of Health Practitioners. PLoS ONE.

[8] Von Steinbüchel N., Wilson L., Gibbons H., Hawthorne G., Höfer S., Schmidt S., Bullinger M., Maas A., Neugebauer E., Powell J. (2010). Quality of Life after Brain Injury (QOLIBRI): Scale Development and Metric Properties. J. Neurotrauma.

[9] Katerndahl D.A. (2008). Impact of Spiritual Symptoms and Their Interactions on Health Services and Life Satisfaction. Ann. Fam. Med..

[10] Sulmasy D.P. (2002). A Biopsychosocial-Spiritual Model for the Care of Patients at the End of Life. Gerontol..

[11] World Health Organization (2001). International Classification of Functioning, Disability and Health: ICF.

[12] Threats T.T., Worrall L. (2004). Classifying communication disability using the ICF. Adv. Speech Lang. Pathol..

[13] Laureys S., Celesia G.G., Cohadon F., Lavrijsen J., León-Carrión J., Sannita W.G., Sazbon L., Schmutzhard E., Von Wild K.R., Zeman A. (2010). Unresponsive wakefulness syndrome: A new name for the vegetative state or apallic syndrome. BMC Med..

[14] Giacino J.T., Ashwal S., Childs N., Cranford R., Jennett B., Katz D.I., Kelly J.P., Rosenberg J.H., Whyte J., Zafonte R.D. (2002). The minimally conscious state: Definition and diagnostic criteria. Neurology.

[15] Bruno M.-A., Vanhaudenhuyse A., Thibaut A., Moonen G., Laureys S. (2011). From unresponsive wakefulness to minimally conscious PLUS and functional locked-in syndromes: Recent advances in our understanding of disorders of consciousness. J. Neurol..

[16] Schiff N.D. (2015). Cognitive Motor Dissociation Following Severe Brain Injuries. JAMA Neurol..

[17] Claassen J., Doyle K., Matory A., Couch C., Burger K.M., Velazquez A., Okonkwo J.U., King J.-R., Park S., Agarwal S. (2019). Detection of Brain Activation in Unresponsive Patients with Acute Brain Injury. N. Engl. J. Med..

[18] Jöhr J., Aureli V., Meyer I., Cossu G., Diserens K. (2022). Clinical Cognitive Motor Dissociation: A Case Report Showing How Pitfalls Can Hinder Early Clinical Detection of Awareness. Brain Sci..

[19] Lin K., Wroten M. (2021). Ranchos Los Amigos. StatPearls [Internet].

[20] Rappaport M., Hall K.M., Hopkins K., Belleza T., Cope D.N. (1982). Disability rating scale for severe head trauma: Coma to community. Arch. Phys. Med. Rehabil..

[21] Krch D., Lequerica A.H. (2017). The factor structure of the Disability Rating Scale in individuals with traumatic brain injury. Disabil. Rehabil..

[22] COMBI The Center for Outcome Measurment in Brain Injur-Disability Rating Scale. https://www.tbims.org/combi/drs/.

[23] Viosca E., Martínez J.L., Almagro P.L., Gracia A., González C. (2005). Proposal and Validation of a New Functional Ambulation Classification Scale for Clinical Use. Arch. Phys. Med. Rehabil..

[24] Jennett B., Bond M. (1975). Assessment of outcome after severe brain damage: A Practical Scale. Lancet.

[25] Fayol P., Carrière H., Habonimana D., Preux P.-M., Dumond J.-J. (2004). Version française de l’entretien structuré pour l’échelle de devenir de Glasgow (GOS): Recommandations et premières études de validation. Ann. Réadapt Médecine Phys..

[26] Cieza A., Brockow T., Ewert T., Amman E., Kollerits B., Chatterji S., Üstün T.B., Stucki G. (2002). Linking Health-Status Measurements to the International Classification of Functioning, Disability and Health. J. Rehabil. Med..

[27] Cieza A., Geyh S., Chatterji S., Kostanjsek N., Üstün B., Stucki G. (2005). ICF linking rules: An update based on lessons learned. J. Rehabil. Med..

[28] Gorbunova A., Zeldovich M., Voormolen D.C., Krenz U., Polinder S., Haagsma J.A., Hagmayer Y., Covic A., Real R.G.L., Asendorf T. (2020). Reference Values of the QOLIBRI from General Population Samples in the United Kingdom and The Netherlands. J. Clin. Med..

[29] Nakase-Richardson R., Whyte J., Giacino J.T., Pavawalla S., Barnett S.D., Yablon S.A., Sherer M., Kalmar K., Hammond F.M., Greenwald B. (2012). Longitudinal Outcome of Patients with Disordered Consciousness in the NIDRR TBI Model Systems Programs. J. Neurotrauma.

[30] Dever A., Powell D., Graham L., Mason R., Das J., Marshall S.J., Vitorio R., Godfrey A., Stuart S. (2022). Gait Impairment in Traumatic Brain Injury: A Systematic Review. Sensors.

[31] U.S. Food and Drugs Administration Public Workshop: Evaluating Inclusion and Exclusion Criteria in Clinical Trials. The National Press Club 2018. https://www.fda.gov/media/134754/download.

[32] Dikmen S.S., Machamer J.E., Powell J.M., Temkin N.R. (2003). Outcome 3 to 5 years after moderate to severe traumatic brain injury. Arch. Phys. Med. Rehabil..

[33] Stocchetti N., Zanier E.R. (2016). Chronic impact of traumatic brain injury on outcome and quality of life: A narrative review. Crit. Care.

[34] Edlow B.L., Claassen J., Schiff N.D., Greer D.M. (2020). Recovery from disorders of consciousness: Mechanisms, prognosis and emerging therapies. Nat. Rev. Neurol..

[35] Rasmussen M.S., Andelic N., Pripp A.H., Nordenmark T.H., Soberg H.L. (2021). The effectiveness of a family-centred intervention after traumatic brain injury: A pragmatic randomised controlled trial. Clin. Rehabil..

[36] Hawthorne G., Gruen R.L., Kaye A.H. (2009). Traumatic Brain Injury and Long-Term Quality of Life: Findings from an Australian Study. J. Neurotrauma.

[37] Gasquoine P.G. (2015). Blissfully unaware: Anosognosia and anosodiaphoria after acquired brain injury. Neuropsychol. Rehabil..

